# Implementing the European guidelines for cardiovascular disease prevention in the primary care setting in Cyprus: Lessons learned from a health care services study

**DOI:** 10.1186/1472-6963-8-148

**Published:** 2008-07-16

**Authors:** Theodora Zachariadou, Henri EJH Stoffers, Costas A Christophi, Anastasios Philalithis, Christos Lionis

**Affiliations:** 1 Nicosia Health Centre, Nicosia, Cyprus; 2 Department of General Practice, School of Public Health and Primary Care (CAPHRI), Maastricht University, Maastricht, The Netherlands; 3 Cyprus International Institute for the Environment and Public Health in association with Harvard School of Public Health, Nicosia, Cyprus; 4 Department of Social Medicine, School of Medicine, University of Crete, Heraklion, Greece; 5 Department of Social and Family Medicine, School of Medicine, University of Crete, Heraklion, Greece

## Abstract

**Background:**

Recent guidelines recommend assessment and treatment of the overall risk for cardiovascular disease (CVD) through management of multiple risk factors in patients at high absolute risk. The aim of our study was to assess the level of cardiovascular risk in patients with known risk factors for CVD by applying the SCORE risk function and to study the implications of European guidelines on the use of treatment and goal attainment for blood pressure (BP) and lipids in the primary care of Cyprus.

**Methods:**

Retrospective chart review of 1101 randomly selected patients with type 2 diabetes mellitus (DM2), or hypertension or hyperlipidemia in four primary care health centres. The SCORE risk function for high-risk regions was used to calculate 10-year risk of cardiovascular fatal event. Most recent values of BP and lipids were used to assess goal attainment to international standards. Most updated medications lists were used to compare proportions of current with recommended antihypertensive and lipid-lowering drug (LLD) users according to European guidelines.

**Results:**

Implementation of the SCORE risk model labelled overall 39.7% (53.6% of men, 31.3% of women) of the study population as high risk individuals (CVD, DM2 or SCORE ≥5%). The SCORE risk chart was not applicable in 563 patients (51.1%) due to missing data in the patient records, mostly on smoking habits. The LDL-C goal was achieved in 28.6%, 19.5% and 20.9% of patients with established CVD, DM2 (no CVD) and SCORE ≥5%, respectively. BP targets were achieved in 55.4%, 5.6% and 41.9% respectively for the above groups. There was under prescription of antihypertensive drugs, LLD and aspirin for all three high risk groups.

**Conclusion:**

This study demonstrated suboptimal control and under-treatment of patients with cardiovascular risk factors in the primary care in Cyprus. Improvement of documentation of clinical information in the medical records as well as GPs training for implementation and adherence to clinical practice guidelines are potential areas for further discussion and research.

## Background

Cardiovascular disease (CVD) is the major cause of premature death in most European populations [1]; it is an important source of disability and contributes to the escalating costs of health care [2]. In Cyprus, CVD accounts for 41% of all annual deaths with slight decline in mortality over the last decade [3]. Recent European guidelines stress the importance of integrated cardiovascular risk prediction in planning preventive strategies [1]. The first and most widely used risk prediction tools were developed with data from the Framingham Heart Study [4]. However, several studies have shown that when Framingham risk charts, derived from a northern American population, are applied to European populations they tend to overestimate the risk of an event [5-7]. In 2003, the European guidelines on CVD prevention, recommended the SCORE (Systematic Coronary Risk Evaluation) risk model as a tool in everyday practice [1]. The SCORE system was developed by the SCORE project group [8] who assembled a pool of datasets from 12 European cohort studies in order to calculate separate risk functions for high and low risk regions of Europe. One of the advantages of using the SCORE database for risk assessment is that it can be easily adapted to national conditions, resources and priorities and takes into account the heterogeneity in CVD mortality across European populations [1]. Furthermore it is a relevant tool to general practice where most primary prevention of Coronary Heart Disease (CHD) and management of people with risk factors take place.

Recently the updated version of the European Guidelines on CVD prevention was issued by the Fourth Joint Task Force [9] redefining priorities and objectives on the prevention of CVD.

In Cyprus, a new National Health System (NHS) will be introduced at the end of 2008. Among other tasks, GPs will have a major role in the management of patients with chronic diseases as well as in the development of prevention strategies in order to promote the well being of patients with high risk behaviours and of the population in general. In the context of this reform and prior to the introduction of the NHS a collaborative project was developed with the Clinic of Social and Family Medicine of the University of Crete. One of the goals was to improve the performance of General Practitioners (GPs) in the management of patients with chronic diseases through training and research programs.

The aim of this study which was part of the aforementioned collaboration was to assess the proportion of high risk patients by applying the SCORE risk model in a cohort of patients with known risk factors for CVD in four Primary Care Health Centres in Cyprus. Another objective was to describe the implications of these guidelines for high risk groups with regard to goal attainment of blood pressure and lipids and to compare recommended and current use of antihypertensives and/or lipid lowering drugs.

## Methods

This study was a retrospective cohort analysis of the medical records of selected patients in four Primary Care Health Centres (PCHCs) in Cyprus. The PCHCs enrolled in the study – two urban and two rural – were located in the Nicosia area and were selected using specific selection criteria. Selection was based on the size of the population served by each PCHC, the opening hours of the PCHC, the number of GPs as well as their postgraduate education and years of experience in primary healthcare (PHC) and the number of nurses and administrative staff at each PCHC.

### Study participants

Of the 9504 charts available, 4633 patients with the diagnosis of type 2 diabetes mellitus (DM2), or hypertension or hyperlipidemia were identified. We made a random selection of 1101 patients and reviewed their medical records in order to assess provided care during the year 2002. Patients less than 18 years of age and those who had no visits during the year examined were excluded from the study.

### Definitions

Hypertension was considered present if the diagnosis hypertension was documented in the medical file, or if the patient was on antihypertensive medication or if there were recordings of at least two consecutive measurements of Systolic Blood Pressure (SBP) ≥140 mmHg and/or Diastolic Blood Pressure (DBP) ≥90 mmHg in the medical records within the year examined [10].

DM2 was considered present if the diagnosis of DM2 was registered in the medical record or by documentation of oral hypoglycemic drugs or combination of oral hypoglycemic drugs and insulin in the medication list or if a fasting plasma glucose (FPG) level ≥126 mg/dl (7.0 mmol/l) was found [11]. FPG values were provided by the Clinical Biochemistry Department of Nicosia General Hospital.

Cardiovascular disease (CVD) was defined as the presence of coronary heart disease (history of myocardial infarction, ischemic heart disease, angina pectoris or surgical history of either coronary artery bypass grafting or percutaneous transluminal coronary angioplasty) or a major or minor stroke or peripheral artery disease [1].

Hyperlipidemia was considered present if the diagnosis of hyperlipidemia was registered in the medical file, or if patients were on lipid-lowering drugs (LLD) or if there was at least one value of Total Cholesterol (TC) ≥190 mg/dl (5 mmol/l) and/or a value of LDL-Cholesterol ≥115 mg/dl (3 mmol/l) and/or a measurement of Triglycerides (TG) ≥150 mg/dl (1.7 mmol/l) documented in their medical records within the year examined [1]. Lipid values were provided by the Clinical Biochemistry Department of Nicosia General Hospital. The biochemistry department uses daily internal controls and participates in three external quality assessment programs.

Medical diagnoses were coded according to the International Classification of Diseases, version 10 (ICD-10) [12] and medications were coded using the fifth level of the Anatomical Therapeutic Chemical system (ATC), version 2000 [13].

Information regarding smoking status of the patients was not documented in the medical files and was obtained through an interview questionnaire performed by the ten trained nurses of the participating PCHCs. Patients were categorized as 'current smoker', 'former smoker' or 'non-smoker'.

### Patient risk stratification

The cardiac risk was evaluated through the "SCORE risk function" according to the Third Joint Task Force European guidelines on CVD prevention [1] for high risk regions, as Cyprus, although a Mediterranean country is classified as high risk. The chart comprises a table of the following parameters: sex, smoking status, SBP, TC and age. Risk was estimated by rounding a person's age to the nearest shown on the chart, their TC level to the nearest whole unit and their SBP to the nearest multiple of 20 mmHg [8]. Risk was defined in terms of absolute probability of developing a fatal cardiovascular event within 10 years and the threshold for high risk was defined as ≥5%. Patients in whom the algorithm could not be applied due to missing data on several risk factors (i.e. lipids, BP and smoking) were excluded from SCORE calculation and from further analysis on goal attainment. Patients were categorized according to the priorities defined in the European guidelines for CVD prevention [1,9] into the following risk groups: (1) patients with CVD (+/- DM); (2) patients with DM (no CVD); 3) patients at high risk to develop CVD (no CVD, no DM, SCORE ≥5%); (4) patients with a low risk to develop CVD (no CVD, no DM, SCORE <5%); (5) patients with missing data for risk calculation.

### Attainment goals for risk factors

To assess attainment goals for risk factors, the most recent SBP and DBP, TC, LDL-cholesterol (LDL-C), HDL-cholesterol (HDL-C) and TG values recorded in the medical records were used. These were compared with the recommendations in the European guidelines [1]. For patients with DM2, FBG (fasting blood glucose) and HbA_1c _(glycated haemoglobin) values were additionally used.

### Medical management

The most updated medication lists were used for analysis of management effectiveness. According to the European guidelines, those patients with a 10 year risk ≥5% of having a fatal CVD event together with SBP ≥140 mmHg and/or TC ≥190 mg/dl (5 mmol/l) and/or LDL-C ≥115 mg/dl (3 mmol/l) should take antihypertensive and/or LLD and aspirin treatment [1]. For the high risk patients (SCORE risk categories 1, 2, and 3) we compared current with recommended treatment patterns for BP and lipids according to the European guidelines. We calculated a delta (Δ) percentage, which expresses the difference between recommended and observed current drug use. A positive delta percentage indicates that the recommended use exceeds current use.

### Statistical analysis

Descriptive analyses were used to evaluate the characteristics of the total study sample. Continuous variables are presented as means with standard deviations (SD) and categorical variables as percentages. T-test was performed to test for differences on continuous variables between groups. All analyses were conducted using SAS (SAS Institute Inc.) version 9.1. P-values less than 0.05 were considered significant.

#### Ethics

Before initiation, the Ethics Committee of Cyprus approved the study [14].

## Results

### Participants' characteristics

The characteristics of the patients enrolled in the study are shown in Table 1. Women represented 62.4% of the overall population (n = 687). DM2 was present in 26.8% of the patients (n = 295). Hypertension and hyperlipidemia were present in 641 (79.5%) and 313 (38.8%) non-diabetic patients respectively. Smoking status was known in 364 patients (33.1%).

**Table 1:** Characteristics of the study population (N = 1101)

**Characteristic**	**Men (n = 414)** **N (%)**	**Women (n = 687)** **N (%)**	**Total (N = 1101)** **N (%)**
	Mean ± SD		
Age (years)	67 ± 10.7	67 ± 10.6	67 ± 11
			
***Age groups (years)***			
≤64	158 (38.2)	276 (40.2)	434 (39.4)
≥65	255 (61.6)	410 (59.7)	665 (59.5)
Missing	1 (0.2)	1 (0.1)	2 (0.2)
			
***Diagnoses***			
• DM2	134 (32.4)	161 (23.4)	295 (26.8)
• Hypertension	307 (74.2)	530 (77.1)	837 (76.0)
• Hyperlipidemia	144 (34.8)	264 (38.4)	408 (37.1)
			
***SCORE risk category***			
1. CVD+/- DM2*	68 (16.4)	35 (5.1)	103 (9.4)
2. DM2 no CVD	114 (27.5)	146 (21.3)	260 (23.6)
3. High risk**	40 (9.7)	34 (4.9)	74 (6.7)
4. Low risk^†^	11 (2.7)	90 (13.1)	101 (9.2)
5. Unclassified risk status^‡‡^	189 (45.7)	374 (54.4)	563 (51.1)
			
***Smoking status***			
• Current smokers	31 (7.5)	10 (1.5)	41 (3.7)
• Former smokers	64 (15.5)	11 (1.6)	75 (6.8)
• Non-smokers	52 (12.6)	196 (28.5)	248 (22.5)
• Missing smoking status	267 (64.5)	470 (68.4)	737 (66.9)

CVD*: definitions see text; DM2: Type 2 Diabetes Mellitus

**Patients with neither CVD nor DM2 but with multiple risk factors resulting in a 10 year risk of ≥5% for developing a fatal CVD event; Patients with TC ≥320 mg/dl (≥8 mmol/l) or LDL ≥240 mg/dl (≥6 mmlo/l) or BP ≥180/110 mmHg.

^†^Patients with neither CVD nor DM2 but with multiple risk factors resulting in a 10 year risk of <5% for developing a fatal CVD event.

^‡‡ ^Patients with neither CVD nor DM2 but with missing data on risk factors for score calculation

### Implementation of SCORE risk chart

The SCORE risk chart was applicable to 157 patients with multiple risk factors, whereas 563 patients (51.1%) were unclassifiable [45.6% of men (n = 189), 54.4% of women (n = 374)] due to missing data mostly on smoking habits as shown in Table 1. Of patients with available data for SCORE calculations, approximately 87% of men and 58% of women aged 60 were classified as high risk, whereas 101 patients (18.8%) from those with available data for SCORE calculation had 10-year risk for fatal CVD event <5%.

### Goal attainment and patterns of medication use in primary and secondary prevention subgroups

As shown in Table 2, the LDL-C goal <100 mg/dl (2.5 mmol/l) was achieved in 28.6% of patients with CVD, 19.5% of patients with DM2 and 20.9% of those with multiple risk factors for CVD. When we applied the more stringent recommendations of the 2007 European guidelines [9], only 5% of the patients with CVD and 8% of DM2 patients had LDL-C <80 mg/dl.

**Table 2:** Goal attainment of blood pressure and lipids in high-risk patients according to the European guidelines with respective mean values.

**Parameters**	Mean ± SD	**Number of patients (%)**
**SCORE RISK CATEGORY 1 (CVD; n = 103)**		
TC (mg/dl), n = 42	207 ± 32.6	
• <175 mg/dl (4.5 mmol/l)		7 (16.7)
• ≥175 mg/dl		35 (83.3)
• missing (% of total N)		61 (59.2)
LDL-C (mg/dl), n = 21	121 ± 36.9	
• <100 mg/dl (2.5 mmol/l)		6 (28.6)
• ≥100 mg/dl		15 (71.4)
• missing (% of total N)		82 (79.6)
BP (mmHg), n = 83	132 ± 15.2 (SBP)	80 ± 6.6 (DBP)
• <140/90 mmHg		46 (55.4)
• ≥140/90 mmHg		37 (44.6)
• missing (% of total N)		20 (19.4)
		
**SCORE RISK CATEGORY 2 (DM2, no CVD; n = 260)**		
TC (mg/dl), n = 136	216 ± 43.2	
• <175 mg/dl (4.5 mmol/l)		23 (16.9)
• ≥175 mg/dl		113 (83.1)
• missing (% of total N)		124 (47.7)
LDL-C (mg/dl), n = 77	127 ± 36.0	
• <100 mg/dl (2.5 mmol/l)		15 (19.5)
• ≥100 mg/dl		62 (80.5)
• missing (% of total N)		183 (70.4)
BP (mmHg), n = 213	138 ± 15.8 (SBP)	82 ± 7.4 (DBP)
• <130/80 mmHg		12 (5.6)
• ≥130/80 mmHg		201 (94.4)
• missing (% of total N)		47 (18.1)
HbA_1c _(%), n = 31	7.2 ± 1.3	
• <7%		12 (38.7)
• ≥7%		19 (61.3)
• missing (% of total N)		229 (88.1)
		
**SCORE RISK CATEGORY 3 (SCORE ≥5%; n = 74)**		
TC (mg/dl), n = 71	241 ± 76.3	
• <175 mg/dl (4.5 mmol/l)		8 (11.3)
• ≥175 mg/dl		63 (88.7)
• missing (% of total N)		5 (6.8)
LDL-C (mg/dl), n = 43	144 ± 49.6	
• <100 mg/dl (2.5 mmol/l)		9 (20.9)
• ≥100 mg/dl		34 (79.1)
• missing (% of total N)		33 (43.5)
BP (mmHg), n = 74	142 ± 20.0 (SBP)	82 ± 9.1 (DBP)
• <140/90 mmHg		31 (41.9)
• ≥140/90 mmHg		43 (58.1)
• missing (% of total N)		0 (0)

TC: Total Cholesterol; LDL-C: Low Density Lipoprotein Cholesterol: BP: Blood Pressure, SBP: Systolic Blood Pressure, DBP: Diastolic Blood Pressure, HbA_1c_: Glycated hemoglobin

BP targets were achieved in 55.4%, 5.6% and 41.9% respectively. Patterns of the gap between recommended and current drug use are shown in Table 3. All delta percentages were positive for the three groups, indicating under prescription of medication. The smallest gap was observed for antihypertensive medication in patients with CVD (Δ = 3.6%), the largest gap was observed among non-diabetic high-risk patients for aspirin use (Δ = 89.2 %).

**Table 3:** Proportion of high risk patients on treatment as recommended by the European guidelines^†^. Proportion of low risk patients and of unclassified patients on treatment.

***SCORE risk category *** ***Treatment***	***(1) CVD (n=103)***		***(2) DM2 (n=260)***		***(3) SCORE ≥5% (n=74)***		***(4) SCORE <5% (n=101)***	***(5) Unclassified(n=563)***
					
	***N (%)***	***Δ(%)***	***N (%)***	***Δ(%)***	***N (%)***	***Δ(%)***	***N (%)***	***N (%)***
**ANTIHYPERTENSIVES**		**3.6**		**13.1**		**6.7**		
• Current	34 (41.0)		86 (40.4)		38 (51.4)		71 (70.3)	382 (67.9)
• Recommended	37 (44.6)		114 (53.5)		43 (58.1))		-	-
								
**LLD**		**30.2**		**38.9**		**47.9**		
• Current	18 (41.9)		50 (36.8)		27 (38.0)		46 (45.5)	173 (30.7)
• Recommended	31 (72.1)		103 (75.7)		61 (85.9)		-	-
								
**ASPIRIN**		**53.4**		**87.7**		**89.2**		
• Current	48 (46.6)		32 (12.3)		8 (10.8)		25 (24.8)	13(2.3)
• Recommended	103 (100)		260 (100)		74 (100)		-	-

SCORE risk categories:

(1): Patients with CVD +/- DM2

(2): Patients with DM2 (no CVD)

(3): Patients with neither CVD nor DM2 but with multiple risk factors resulting in a 10 year risk of ≥5% for developing a fatal CVD event; Patients with TC ≥320 mg/dl (≥8 mmol/l) or LDL ≥240 mg/7 dl (≥6 mmlo/l) or BP ≥180/110 mmHg.

(4): Patients with neither CVD nor DM2 but with multiple risk factors resulting in a 10 year risk of <5% for developing a fatal CVD event.

(5): Patients with neither CVD nor DM2 but with missing data on risk factors for score calculation

^†^Values are for those patients with available data

## Discussion

### Main findings

This was the first study in Cyprus on cardiovascular risk stratification by means of the SCORE system according to the European guidelines. The implementation of this risk model in a selected cohort of primary care patients with established risk factors for CVD has labelled a substantial proportion as high risk individuals (overall 39.7%). Almost 90% of men aged 55 and 57.8% of women aged 60 were classified as high risk. In a similar study by Getz et al [15], 91.4% of men aged 55 and 57.3% of women at age 60 were classified as high risk according to SCORE chart, findings that are in accordance with those of our study.

In addition, this study demonstrates a gap between recommended and current drug use for the high risk population. Two studies from Norway [16,17] found that 42% and 52% of men aged 45–64 respectively would be candidates for antihypertensive or LLD treatment, whereas according to another study [18], 29% of dyslipidemic patients should receive LLD. In our study, the gap between recommended and current antihypertensive use was larger in the diabetic group of patients (13.1%). The fact that only 42% of patients with CVD were taking LLD and even less (29%) achieved recommended LDL-C is a matter of concern. In a study from Spain [19], 65% of patients with CHD were on LLD and 31% of those achieved LDL-C target whereas in a retrospective study by Straka et al [20], 71% of patients with CHD were on LLD and 35% of those had LDL <100 mg/dl. Harz et al [21] showed that 50% of patients with CHD were on LLD and one in three reached lipid goal. According to the results of EUROASPIRE [22] I and II, 54% of patients with established CHD had BP off target (≥ 140/90 mmHg) as compared to 44.6% of our study patients of the similar group.

Regarding the diabetic population of the study 19.5% achieved their LDL-C goal. In other studies [23-25] these percentages ranged from 16.5% to 53%, whereas in a Greek study [26] 17.5% of diabetic patients achieved LDL-C <100 mg/dl. BP control was particularly low among patients with DM2 (7.5%) as compared to other studies [23,25,27-29], with percentages ranging between 10.3% and 35%. Women with DM2 had worse mean FBG (160 vs 140 mg/dl, p = 0.044) as compared to diabetic men, a findings also supported by an Italian study [25].

### Limitations

Several limitations of our study should be noted. This study examined a selected population of patients with already known risk factors for CVD (hypertension, hyperlipidemia, DM2) and was not performed in healthy individuals. Thus, our findings are restricted to this clinical segment of the population. Also, the study was performed in only four PCHCs from the total of fifteen in Nicosia area and in only one area of the island. Results must thus, be interpreted with caution and cannot be generalised for the entire population of the island.

Furthermore, many of the patients were already receiving antihypertensive or LLD treatment which could have modified their risk profile at the time of their risk assessment. In addition, the SCORE risk chart could not be calculated in over 50% of the participants due to underreporting of clinical information in the medical files. As a result, the 10-year risk for a fatal cardiovascular event is unknown for more than half of the studied patients and therefore we can not draw conclusion for the remaining population of the study.

### Implications of the study to national health care policy

There are some important messages derived from this study that should convey to health policy makers who are responsible for the design and implementation of the new NHS in Cyprus. The fact that GPs underreport clinical information (smoking behaviour, lipid values and BP) is indeed a matter of concern. It should be stressed out that a major step in improving the management of CVD risk factors is to accurately report them and treat them. However, there have not been any studies so far in the PHC setting of Cyprus to assess the level of documentation of clinical information by the GPs. We can either assume that GPs do perform blood tests, measure BP and ask about the smoking habits of their patients but they do not document this information in the medical files or that they simply do not perform them. These however, are only assumptions and further studies are needed in order to reach safe conclusion. Also, as shown from our results, GPs do not follow clinical practice guidelines in their everyday practice for the management of patients with common chronic conditions. This was indicated from the under-treatment of high risk patients as well as from gaps in goal attainment. Potential barriers could be administrative, educational and time pressures. These issues could be addressed through introduction of organisational systems to support the management of patients with chronic diseases (i.e. registries, computerised recall and reminder systems) as well as effective charting systems (e.g. flow sheets) to improve quality assurance and documentation [29].

Furthermore, our study was performed before the publication of the recent European guidelines [1,9] and therefore GPs were unaware of the recommendations stated in the guidelines. However, our intention was to have an initial evaluation of the care delivered in the PHC setting of Cyprus in order to identify failures and stimulate for actions before the introduction of the new NHS. This study served as the first part of a quality project that was followed by the implementation of a multifaceted intervention in DM2 patients.

Finally, the fact that Cyprus is included in the high risk regions for CVD and whether these guidelines overestimate risk in the general population are matters that should raise discussion. So far, there are no national guidelines for CVD prevention and only recently the Cyprus College of Cardiology adapted the Greek version of the European guidelines. Until epidemiological studies are performed addressing the above mentioned issues, including the assessment of the risk level of the population at risk for CVD, current guidelines could and should serve as a tool to GPs for prioritising patients in order to address the role of lifestyle changes, the management of cardiovascular risk factors and the use of other prophylactic drug therapies in the prevention of clinical CVD.

## Conclusion

Implementation of the European guidelines in patients with risk factors for CVD in the Primary Care in Cyprus has classified a substantial proportion at high risk for fatal cardiovascular event and revealed suboptimal quality of care with regard to risk factor registration, medical treatment and achievement of treatment targets. The study results could act as a stimulus to improve Cypriot primary care for cardiovascular disease.

## Authors' contributions

CL conceived the idea of the study, participated in the design of the study and contributed to the draft and final version of the manuscript. TZ designed and coordinated the study and drafted the manuscript. HEJHS participated in the design of the study and contributed to the draft and the final version of the manuscript. CAC performed the statistical analysis. AP contributed to the final version of the manuscript. All authors read and approved the final manuscript.

## Pre-publication history

The pre-publication history for this paper can be accessed here:


